# Fournier’s gangrene as initial presentation of rectal cancer

**DOI:** 10.1093/jscr/rjaf915

**Published:** 2025-11-13

**Authors:** Saúl Sánchez Iglesias, Cristina de la Cruz Cuadrado, Julián de Pedro Conal

**Affiliations:** Hospital Universitario de Toledo, Avenida del Rio Guadina, Toledo 45007, Spain; Hospital Universitario de Toledo, Avenida del Rio Guadina, Toledo 45007, Spain; Hospital Universitario de Toledo, Avenida del Rio Guadina, Toledo 45007, Spain

**Keywords:** case report, Fournier’s gangrene, rectal cancer

## Abstract

Fournier’s gangrene is a necrotizing fasciitis of the perineum that can occur due to a loss of integrity of the gastrointestinal or urethral mucosa. Its appearance as the debut of a perforated rectal neoplasm is unusual, more uncommon in young patients, leading to an advanced stage of the disease. Therefore, we present the case of a 47-year-old male with Fournier’s gangrene as the debut of a rectal cancer that, despite severe presentation, the pathological stage was early (pT2N0), highlighting the importance of considering neoplastic etiology in perineal gangrene and the need for an early multidisciplinary approach.

## Introduction

Fournier’s gangrene is a necrotizing fasciitis of the perineum that may result from gastrointestinal or urethral mucosal disruption. Its presentation as the first manifestation of perforated rectal cancer is unusual, particularly in young patients, and is generally associated with advanced disease.

## Case report

**Figure 1:**
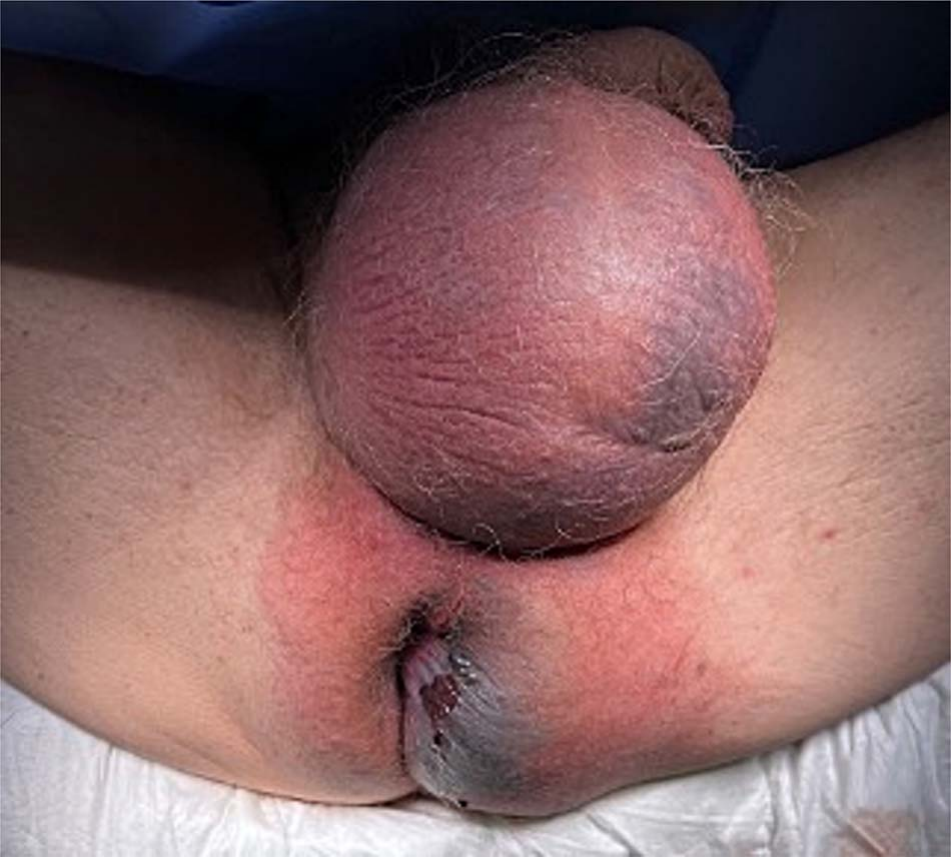
Initial presentation of Fournier’s gangrene.

**Figure 2:**
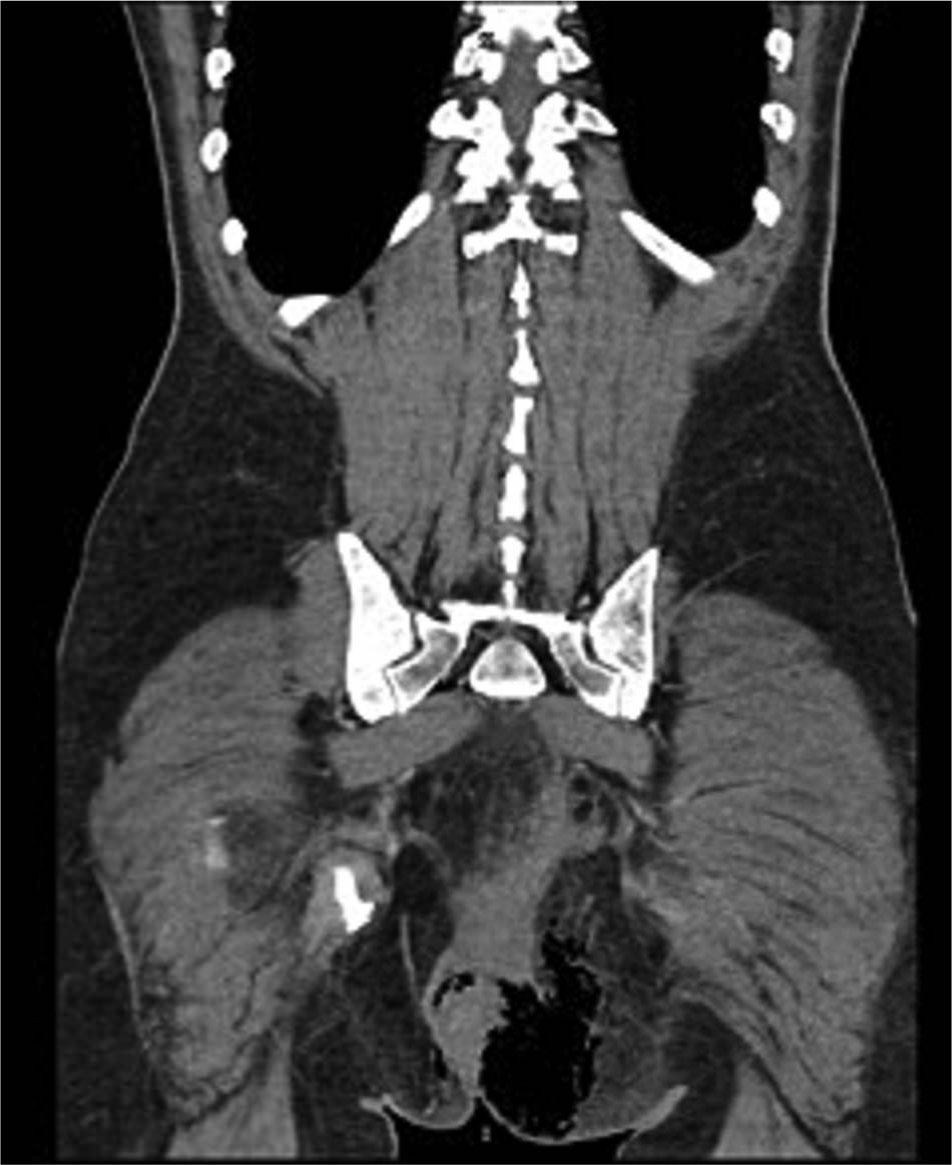
Diagnostic CT scan.

We present the case of a 47-year-old male with no prior medical history who arrived at the Emergency Department with a rapidly evolving perianal abscess (Fig. 1). Within hours, he developed Fournier’s gangrene secondary to an undiagnosed rectal neoplasm (Fig. 2).

**Figure 3:**
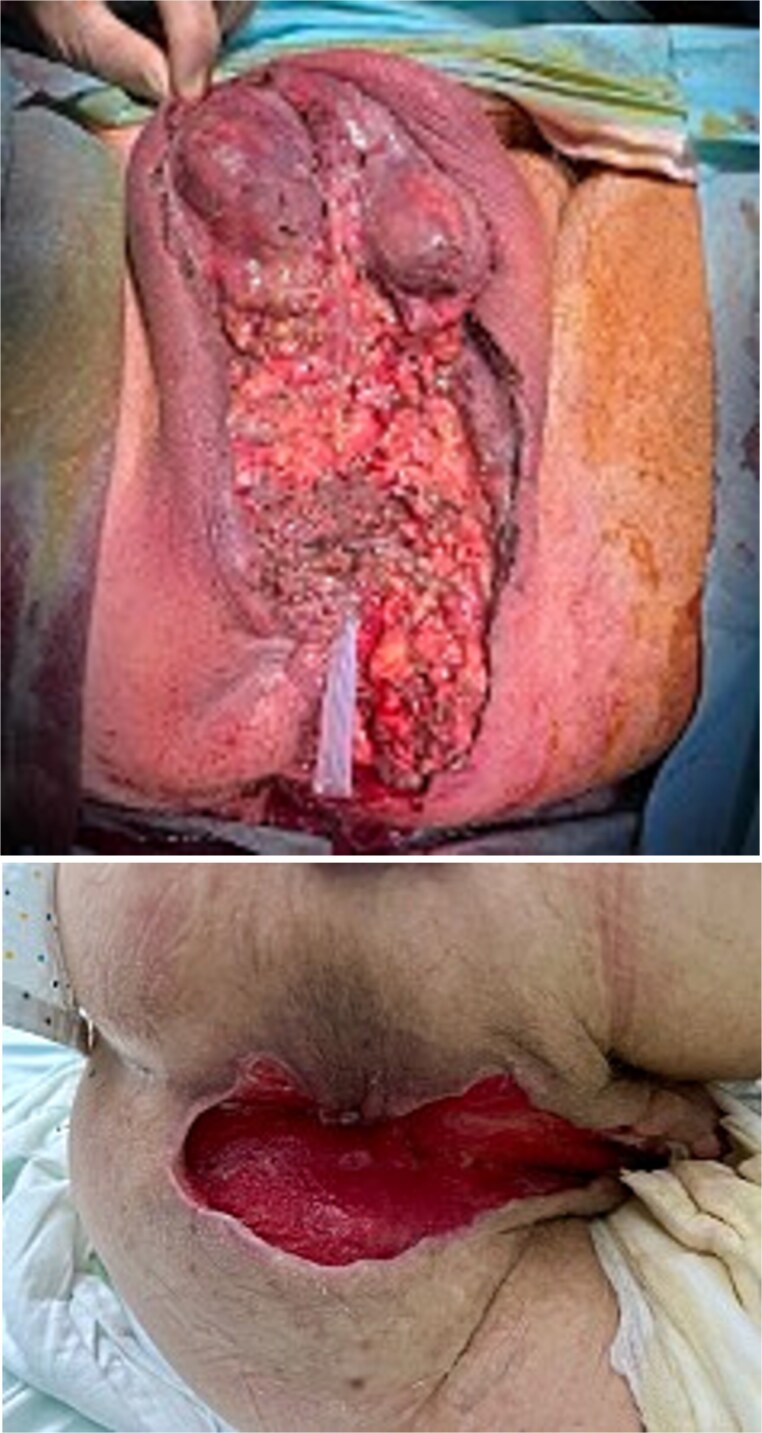
Surgical debridement and early postoperative outcome.

Urgent surgery was performed, including debridement of necrotic tissue, biopsy of the distal rectal lesion, and diverting colostomy. The patient’s postoperative course was favorable (Fig. 3).

**Figure 4:**
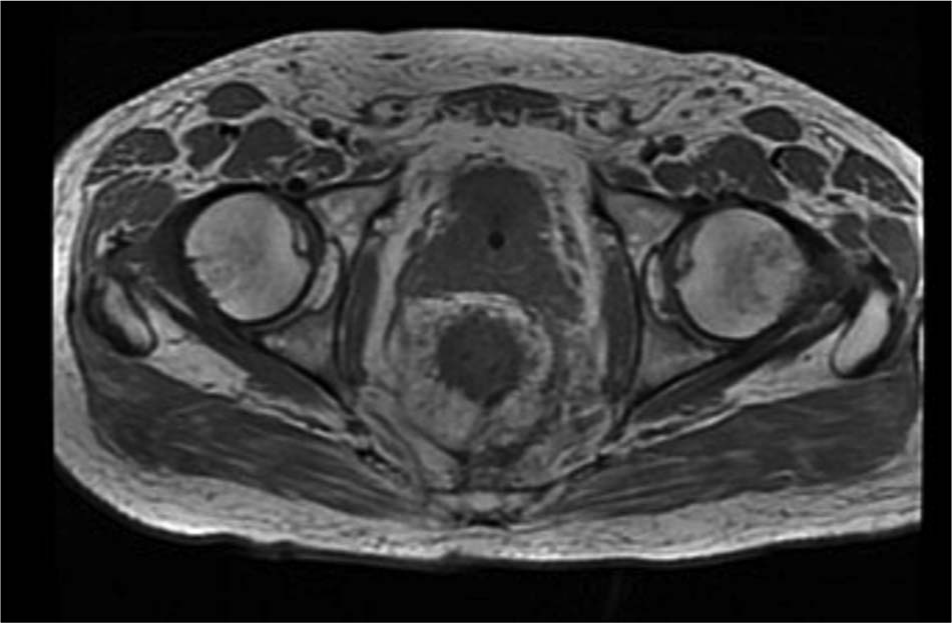
Pelvic MRI staging cT4bN1a.

Histopathological analysis revealed a moderately differentiated adenocarcinoma. Colonoscopy showed no additional lesions and tumour markers were normal. PET-CT excluded distant disease. Pelvic MRI demonstrated involvement of the left levator ani, mesorectal fascia, and suspicious lymph nodes, suggestive of cT4bN1a disease (Fig. 4).

**Figure 5:**
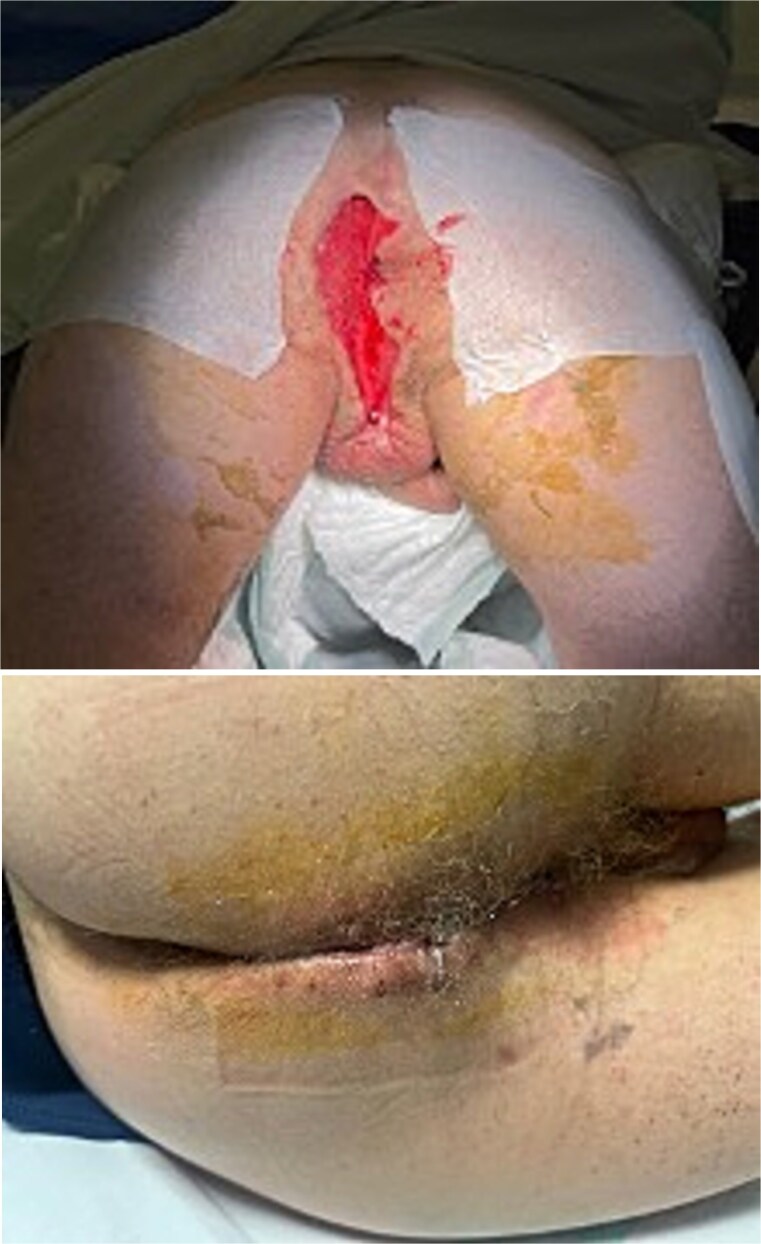
Abdominoperineal resection with favorable perineal healing.

After multidisciplinary team review, the patient underwent abdominoperineal resection with terminal colostomy in the left iliac fossa and perineal closure using a modified Friedrich technique. Postoperative evolution was favourable, with progressive healing of the perineoscrotal wound by secondary intention (Fig. 5).

Final pathology revealed a moderately differentiated adenocarcinoma (G2) without mucinous features, staged pT2 pN0 pMx, with negative surgical margins. Given the early stage, the patient did not require adjuvant therapy and to date has not presented recurrence.

## Discussion

Fournier’s gangrene is a severe surgical emergency characterized by rapidly progressive necrosis of perineal soft tissues, with reported mortality ranging from 20% to 40%, depending on comorbidities and timeliness of treatment. Although most cases are associated with urological or anorectal infections, its presentation as the first manifestation of perforated rectal cancer is extremely rare [1, 2].

Few cases have been described in the literature, most involving advanced tumours and poor outcomes in elderly or comorbid patients [3, 4]. In contrast, our case is notable for involving a young, otherwise healthy patient, whose final tumour staging was unexpectedly early (pT2N0) despite the aggressive clinical debut.

This discrepancy between clinical and pathological staging has been reported previously. Yoshino et al. described a similar case where Fournier’s gangrene due to rectal cancer did not correspond to locally advanced pathology, highlighting the role of early multidisciplinary intervention [3]. Likewise, Hamidian Jahromi *et al*. emphasized the therapeutic challenge of managing severe sepsis alongside an unknown malignancy, requiring prioritization of hemodynamic stabilization, urgent debridement, and targeted biopsies before definitive oncologic surgery [4].

From a surgical standpoint, the staged strategy employed here (initial debridement, urgent fecal diversion, followed by abdominoperineal resection) aligns with current recommendations [5]. This sequence facilitates infection control, reduces risk of tumour dissemination, and prepares the patient for definitive resection under safer conditions [5]. Secondary intention closure of the perineal wound using a modified Friedrich technique also proved effective in this setting [6].

Final histology revealed a moderately differentiated tumour without nodal involvement or metastasis, suggesting that early perforation may have triggered bacterial dissemination and local necrosis without necessarily reflecting aggressive tumour biology. MRI findings of apparent invasion likely represented reactive inflammatory changes rather than true extension, consistent with final pathology.

In conclusion, Fournier’s gangrene should prompt consideration of an underlying neoplastic cause, even in young patients. Early suspicion and histological confirmation allow for tailored oncologic management, avoiding both undertreatment and overtreatment. The limited literature supports structured, multidisciplinary approaches to optimize outcomes in these rare but severe scenarios [3–8].

## Conclusions

Fournier’s gangrene is a surgical emergency due to its fasciocutaneous necrosis and high mortality. It is essential to consider rectal cancer as a possible underlying cause, since prognosis and survival depend on timely and appropriate initial management.
